# Supplementary material for: Oomycete metabarcoding reveals the presence of Lagenidium spp. in phytotelmata
Source: PeerJ. 2019 Oct 14;7:e7903. doi: 10.7717/peerj.7903 (PMC6796956; doi:10.7717/peerj.7903)
Supplement: File S1 [file peerj-07-7903-s001.docx]

**Supplemental information on the plant taxonomic identification:**

A total of four plants were selected for analysis (Fig. 2). These plants were all characterized by a leaf axil structure that allowed for the retention of sampleable volumes of water. Anecdotical observations supported the hypothesis that invertebrates used these sources of water, as several dead and live insects, including mosquito larvae and pupae, were readily pipetted during water sampling (not shown). Taxonomic identification of these plants relied in part on the sequencing of plant barcodes. Sequence fragments corresponding to the chloroplastic *trnH*-*psbA* and the *trnC*-*petN* spacer regions were obtained for all plants. Sequences ranged from 163 to 597 bp, and 403 to 641 bp, for the *trnH*-*psbA* and the *trnC*-*petN* barcodes, respectively, and are available publicly in the GenBank/EMBL/ DDBJ databases under the accession numbers MN099106- MN099113. Homology searches (not shown) identified all plants as members of the family Bromeliaceae, in agreement with tentative taxonomic classifications based on morphological characteristics. Taxonomical identifications at the genus and species levels were not attempted.

**Supplemental information on PacBio sequencing:**

A total of 40,021 PacBio reads totaling 32,436,900 bp were obtained from one SMRT cell. The average number of full pass per reads was 24.62, and the average read length was 810 bp, matching the amplicons expected lengths. The average quality score per insert was measured at 99.69%. Following the removal of inserts that did not include the mirroring barcodes on both ends (51 reads), a stringent QC threshold was used to eliminate low-quality reads.
